# The Role of the RecFOR Complex in Genome Stability

**DOI:** 10.3390/ijms26125441

**Published:** 2025-06-06

**Authors:** Piero R. Bianco

**Affiliations:** Department of Pharmaceutical Sciences, College of Pharmacy, University of Nebraska Medical Center, Omaha, NE 68198-6025, USA; pbianco@unmc.edu

**Keywords:** RecF, RecR, RecO, recombination mediator, genetic recombination, DNA repair, SSB protein

## Abstract

The maintenance of genome stability requires the coordinated actions of multiple proteins and protein complexes. One critical family of proteins is the recombination mediators. Their role is to facilitate the formation of recombinase nucleoprotein filaments on single-stranded DNA (ssDNA). Filament formation can take place on post-replicative ssDNA gaps as well as on 3′-tailed duplexes resulting from helicase–nuclease processing. In prokaryotes, the RecF, O, and R proteins are widely distributed and mediate RecA loading as either the RecFOR or RecOR complexes, depending on the species being studied. In this review, I compare and contrast the available biochemical and structural information to provide insight into the mechanism of action of this critical family of mediators.

## 1. Introduction

**Figure 1:**
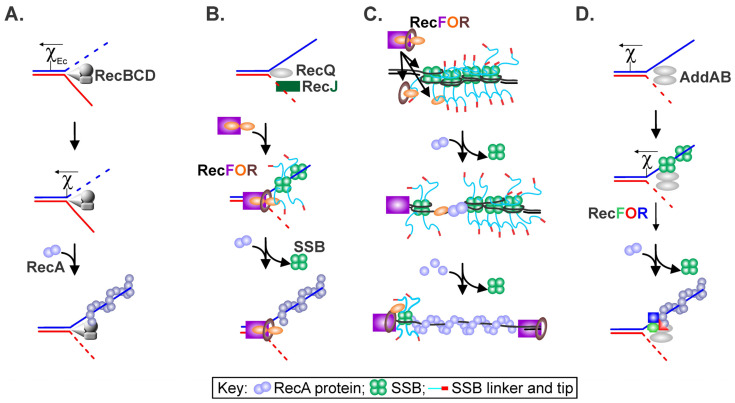
Mechanisms of RecA filament nucleation. (**A**) RecBCD loads RecA in a chi-dependent manner. Once the translocating RecBCD encounters an appropriately oriented chi-sequence (χ_Ec;_ chi = χ = crossover hotspot instigator), it pauses, and the nuclease activity on the 3′-terminated strand is attenuated while the nuclease activity on the 5′-terminated strand is upregulated. Concurrently, the RecA-loading domain is exposed so that RecA protein can be specifically loaded onto the now intact 3′-terminated strand once the χ-modified enzyme continues translocating and unwinding DNA past the chi sequence. (**B**) In the absence of RecBCD, RecQ helicase unwinds the DNA duplex while RecJ degrades the 5′-terminated strand. SSB binds to the exposed 3′-terminated single strand of DNA. RecFOR binds to the DNA (possibly at the ss–dsDNA junction) and RecOR mediates the displacement of SSB concomitant with the loading of RecA. (**C**) The ssDNA in post-replicative gaps is bound by SSB. RecFOR binds to the DNA (possibly at the ss–dsDNA junction) and RecOR mediates displacement of SSB concomitant with the nucleation of an RecA filament. RecFR limits the extension of the RecA filament into the duplex DNA region downstream of the gap. (**D**) In organisms other than *E. coli*, AddAB (or AdnAB) unwinds duplex DNA from an exposed end and simultaneously degrades the 5′-terminated strand, leaving the 3′-terminated strand intact, which is bound by SSB. These helicase/nucleases are not known to load RecA, which is instead facilitated by RecFOR, concomitant with the displacement of SSB.

A critical goal of recombination mediators in bacteria is to facilitate the formation of RecA protein nucleoprotein filaments on single-stranded DNA (ssDNA). These filaments are central to genome stability, are active in DNA recombination and the induction of the SOS response, and are key to SOS mutagenesis [1,2]. In Escherichia coli, the RecBCD enzyme processes double-stranded DNA (dsDNA) breaks and, in a chi-dependent manner, actively loads RecA onto nascent ssDNA, outcompeting the single-strand DNA-binding protein (SSB) (Figure 1A) [3,4,5]. This comprises the early steps of the primary recombination pathway in this organism, i.e., the RecBCD pathway [4,6].

Several years ago, an alternate recombination pathway was identified in *E. coli* that is different at the initiation stages from the primary pathway (Figure 1B,C). In a *recBC sbcBC* background, the RecF, O, and R proteins are necessary for recombination initiation in what is now known as the RecF pathway [7,8]. It was later shown that the RecA, RecJ, RecF, RecN, RecO, RecR, and RecQ proteins are required in addition to the ubiquitous SSB (Figure 1B) [9,10,11]. In this pathway, dsDNA end processing is catalyzed by the RecQ helicase, with nucleolytic processing provided by the RecJ nuclease. Together, these proteins produce a 3′-ssDNA-tailed duplex to which SSB binds [12]. Subsequently, the RecFOR proteins mediate the displacement of SSB concomitant with the loading of RecA onto the ssDNA [13,14]. Displacement by RecO involves interactions between the oligonucleotide–oligosaccharide binding fold (OB-fold) of the mediator and the intrinsically disordered linker of SSB, as well as RecO–SSB acidic tip binding [15,16,17,18]. Even though the RecBCD and RecF pathways are generally considered separate, their parts can interchange, as observed in cells containing an RecA-loading-deficient *recB* mutant. Here, loading requires both RecJ for dsDNA end processing and RecFOR to load RecA [19]. Once homologous pairing by the resulting RecA nucleoprotein filament occurs, the downstream steps of each pathway are the same.

In other bacteria, however, the situation is quite different. In *Bacillus subtilis*, the RecBCD homolog AddAB processes dsDNA ends in a manner analogous to RecBCD, but the enzyme is not known to load RecA [20]. In contrast, *Mycobacteria* possess both RecBCD and a heterodimeric helicase–nuclease known as AdnAB, with the latter enzyme functioning in the primary recombination pathway [21,22,23]. However, AdnAB on its own, is also not known to load RecA. In *Deinococcus*, both RecBCD and AddAB are absent while the genes of the RecFOR pathway are present (*recJ*, *F*, *O*, *R*, and *Q*) [24]. In *Thermus thermophilus*, RecBCD is absent, while dsDNA ends are processed by AddAB, and, again, this enzyme on its own is not known to load RecA [25].

Instead, in both *Bacillus* and *Thermus*, the RecOR proteins facilitate RecA loading onto SSB-coated ssDNA following the action of AddAB, and this may be similar in other organisms where an AdnAB enzyme processes dsDNA ends (Figure 1D) [25,26,27]. In vivo, *Bacillus* RecF enhanced RecA filament nucleation and/or elongation above that seen for RecOR, similar to what was observed in vitro for the *E. coli* proteins [26,28]. It is not surprising then that in bacteria other than *E. coli*, deletion of the *recFOR* genes and RecF pathway members has dramatic effects on viability.

In *E. coli*, the deletion of the *recF*, *O*, and *R* genes is not lethal and, in the right genetic background, renders cells UV-sensitive and defective for plasmid recombination [7,29,30]. In contrast, in *Deinococcus*, deletion of these genes exhibits a phenotype similar to that of Δ*recA* [31]. Furthermore, the inactivation of *recJ* is lethal. This led the authors to propose that the RecFOR proteins are essential for DSB repair through extended synthesis-dependent strand annealing (ESDSA), while the RecJ protein is essential for viability. In *Thermus*, a Δ*recF* strain could not be constructed, suggesting that it might be essential for viability, and deletion of *recO* resulted in spontaneous mutations in the *addA* or *addB* genes [25]. While RecFOR is necessary for DNA repair and chromosome segregation, this pathway is not essential for viability in this organism. In contrast, in *Mycobacteria*, the RecFOR system has a central role in homology-dependent DNA repair, and RecR is essential for all three recombination and DNA repair pathways in this organism [32]. In contrast, in *Campylobacter jejuni* and Helicobacter pylori, *recF* is absent, and RecA loading requires RecOR [33,34].

In *E.coli*, the RecFOR proteins are essential for the induction of the SOS response and the repair of single-stranded DNA gaps [35,36,37]. In other bacteria, the RecFOR proteins facilitate RecA loading onto nuclease-tailed duplexes and ssDNA gaps. These gaps can arise in the genome in several ways [38]. They are often called post-replication or daughter strand gaps, as they form when stalled replisomes disengage and then reinitiate downstream of a site of DNA damage [39]. The RecF, O, and R proteins are essential for RecA filament formation in these gaps (Figure 1C). The requirement for RecFOR arises due to the high affinity of SSB for ssDNA (K_d_ = 1.45 × 10^−7^ to 4.04 × 10^−9^ M) and because SSB and RecA binding are competitive [40,41]. Thus, RecA requires assistance to displace SSB, provided here in the form of the mediator proteins. The nucleation of filament formation on SSB-coated ssDNA is rate-limiting and is accelerated by RecOR, further enhanced when RecF is present, and may involve RecO–SSB, RecA–SSB, and RecA–RecR interactions [14,28,40]. In addition, the RecFR complex binds to ss–dsDNA junctions, with the complex at the 3′-end of the RecA filament preventing extension into the dsDNA downstream of the ssDNA in the gap [42].

While most if not all bacteria contain the RecF, O, and R proteins, there are differences and similarities in their roles in genome stability. Both of these properties contribute to our understanding of the mechanism of action of the RecFOR, RecFR, and RecOR complexes as well as the individual proteins. In this review, I will compare and contrast these complexes as much as possible to provide insight into the mechanism of action of the RecFOR proteins in homologous recombination and DNA repair.

## 2. Sequence Similarity

There is a growing body of data on the RecF, O, and R proteins from *E. coli*, *Bacillus*, *Thermus*, *Deinococcus*, *Caldanaerobacter*, *Campylobacter*, *Pseudomonas*, and *Mycobacteria*. To compare these proteins from different organisms, we thought it prudent to determine how similar they are to one another. To achieve this, sequence alignments of the proteins of the abovementioned organisms were carried out using Promals3D, and images were assembled using ESPript 3 [43,44,45]. The results show that, as expected, RecO proteins are similar to each other, while RecF proteins are similar to each other and RecR proteins are similar to each other (Supplementary Figure S1A–C). The RecR proteins are more similar than the RecF and O proteins, as indicated by the colored regions in the alignments.

For RecF, my analysis is similar to what Koroleva et al. found [46]. That is, the most conserved regions (highlighted in red) correspond to the Walker A and B motifs located at residues 38 and 317, respectively. In addition, the S and D motifs first identified in Rad50 are well conserved in bacterial RecF proteins. These are located at residues 285-87 and 322-24, respectively. Finally, residues comprising the partially buried charge cluster in Lobe II, located at positions 135–140 and amino acid 164, are invariant.

For RecO, the overall alignment is poor (Supplementary Figure S1B). Only glycine 145 is invariant. The cysteine residues responsible for binding zinc, located at positions 53, 156, 173, and 176, are not well conserved. This makes sense, as not all homologs coordinate the metal ion, including that of *E.coli*.

Finally, as alluded to above, RecR is the most well-conserved component of the RecFOR complex [47]. First, the cysteine residues at positions 57, 60, 69, and 72 are invariant. These amino acids coordinate the zinc atom. Second, the TOPRIM domain, i.e., amino acids 80–173, is also well conserved. Third, amino acids involved in binding RecO vary in their conservation. In region I, amino acids 17–23, 4 out of 7 positions are invariant. In region II, amino acids 112–116 and 121 are not well-conserved; region III tolerates conservative substitutions with positions 149 and 150 invariant. Similarly, region IV also tolerates conservative substitution with only two amino acids, while positions 182 and 187 are invariant.

## 3. Structural Similarity

Similar to the information in the primary amino acid sequence alignments, there are multiple crystal structures available for each protein. To determine how similar these are, selected structures were aligned in a pairwise fashion using the structural alignment tool of the PDB, namely the TM-align option [48,49]. These alignments show that the structures of RecF proteins and, separately, RecR proteins, align well, with RMSD values between 1.75 and 2.29 Å, respectively (Table 1 and Supplementary Figure S2). In contrast, the RecO structures do not align as well, with RMSD values between 2.09 and 3.69 Å. The poorest alignment is observed when comparing *E. coli* RecO to other organisms. Here, the differences occur in the following three regions: the loop region where the zinc finger occurs (this is absent in EcRecO), the C-terminus where the SSB C-terminal domain peptide binds, and the N-terminal domain, immediately adjacent to the OB-folds (Supplementary Figure S2, regions 1–3; dashed boxes). The poor structural alignment in the zinc finger region is consistent with the absence of zinc binding in several RecO homologs, with poor sequence conservation in this region of the proteins (Supplementary Figure S1B).

## 4. Protein Domain Organization

**Figure 2:**
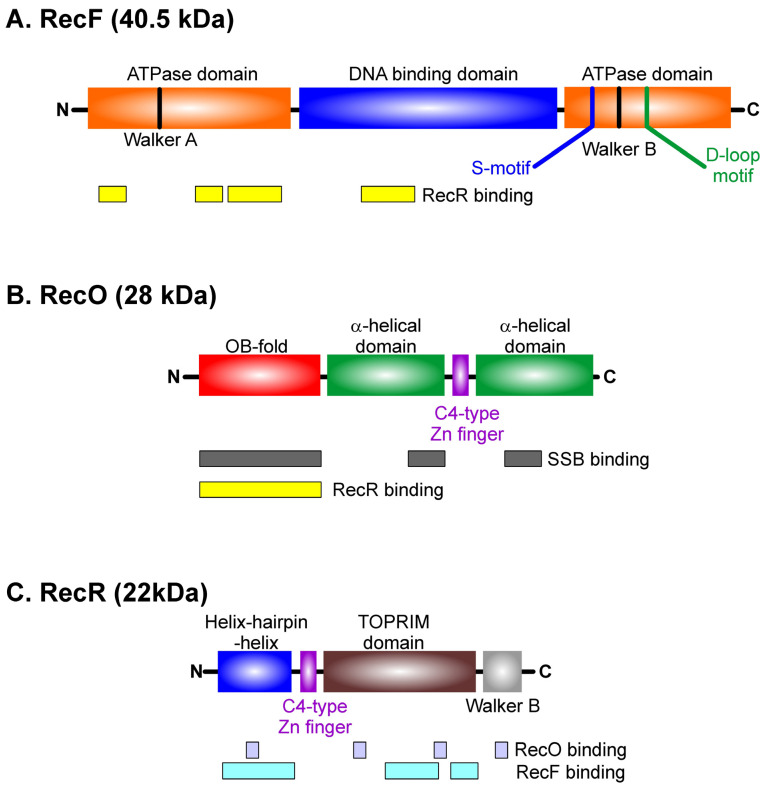
RecF, O, and R protein domain organizations are shared among different bacteria. The schematics for each protein are adapted from [50]. Proteins are presented in order of decreasing size, with the relevant domains color-coded. The regions of each protein responsible for mediating interactions with partners are presented below each schematic. Details are discussed in the text.

The primary and secondary structure analyses above show that the RecF, O, and R proteins from different organisms are sufficiently similar to one another, so a general domain organization should apply. That is, RecF proteins are similar to each other, while RecR proteins are similar to RecR and RecO proteins and to each other. I have used the domain organization from Nirwal et al. and added additional information from the work of others to provide as comprehensive a view as possible (Figure 2) [46,50,51,52].

The RecF protein is comprised of the following three domains: a DNA binding domain sandwiched between two ATPase domains, containing the Walker A and B motifs, respectively. The C-terminal ATPase domain also contains the S- and D-loop motifs, found in the head domain of the eukaryotic Rad50 protein [46,53]. The RecF protein forms a homodimer and also binds to RecR [50,51,54]. The DNA binding domain enables binding to gapped as well as single- and double-stranded DNA [55,56,57]. Binding to both DNA and ATP is modulated by extensive interactions with RecR [58]. RecF is monomeric in solution and, in this form, does not hydrolyze ATP. However, it demonstrates ATP-dependent dimerization and, when bound to DNA, the complex has ATPase activity [46,59]. Here, the Walker A motif functions as a switch playing a central role in both ATP binding and protein dimer formation [59].

The RecO protein consists of three general domains [60,61]. An N-terminal OB-fold followed by two α-helical domains that are split by a C-4 type zinc finger is present in many homologs, but not in the *T. thermophilus* or *E. coli* proteins [61]. RecO proteins exist as monomers in solution. RecO is known to bind SSB [54]. The OB-fold binds to the intrinsically disordered linker of SSB while short peptides corresponding to the last 8–10 residues of SSB bind to a pocket formed between the two α-helical domains [62,63]. Linker/OB-fold binding likely represents the primary binding interaction, as an SSB mutant lacking the last 8 residues does not bind to RecO [16,63]. In addition to binding SSB, the RecO OB-fold also binds to RecR [50]. The complex has a 1:1 stoichiometry, likely as a 2:2 heterotetramer in *E. coli* and as a 4:2 (R:O) heterohexamer in *Deinococcus* [61,64].

RecR is the smallest protein (~22 kDa) and consists of the following four domains: a helix–hairpin–helix (HhH) at the N-terminus, followed by a C4-type zinc finger, a TOPRIM domain, and, finally, a Walker B motif responsible for ATP binding [65,66]. The HhH domain is essential for DNA binding; the zinc finger has a structural role and the TOPRIM domain is required for both RecF and RecO binding, with a preference for RecF over RecO [66,67,68]. Studies with *Thermoanaerobacter tengcongensis* RecR showed that the role of the zinc finger is to stabilize the three-dimensional structure of the protein [69]. RecR exists as either a dimer, tetramer, or octamer in solution, depending on the genus [67,69,70]. As a tetramer, the complex possesses a central cavity, 30–35 Å in diameter, that is sufficiently large enough to accommodate dsDNA with the HhH domains facing inward [50,67,70]. In the absence of DNA, the protein binds to both RecF and RecO as the tetramer with the following stoichiometry: 2:2:4 (F–O–R) [50].

## 5. Higher-Order Structural Insight and Partner Binding

**Figure 3:**
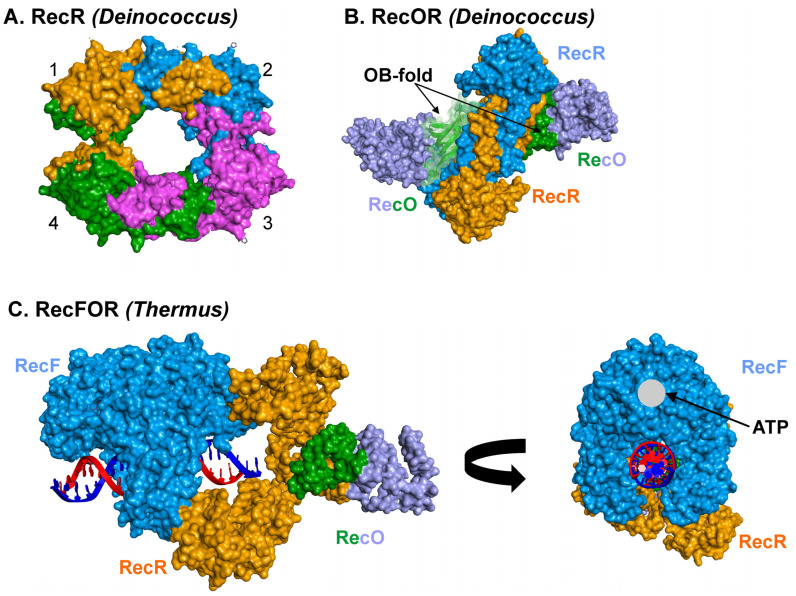
The RecF, O, and R proteins assemble into distinct functional, higher-order structures. Images were generated using Discovery Studio (Biovia). The Connolly surfaces for each protomer are shown [77]. (**A**) RecR can assemble into a tetramer. The four subunits (each shown in a different color) assemble to form a ring with a central hole sufficiently large to accommodate dsDNA. (**B**) The RecOR complex is shown from the side. Two subunits of the RecR tetramer are seen in this view with two RecO monomers bound. Each RecO binds to RecR via its N-terminal OB-fold (colored green). The remaining residues of each ReO are colored mauve to facilitate visualization. (**C**). The RecFOR complex bound to dsDNA is viewed from two angles. Left: the complex is viewed from the side with all protomers of RecF colored blue and RecR colored orange. The N-terminal OB-fold of RecO is colored green, identical to panel (**B**), and is bound to RecR in proximity to the duplex DNA end. Right: the complex is rotated to enable visualization of the interaction of RecF with the DNA. From this angle, the RecF dimer straddles the duplex DNA, anchoring the complex.

RecR protein is the key mediator of RecFOR function as it binds to both RecF and RecO, affecting their activities as RecFR, RecOR, and RecFOR [51,71,72]. In *M. tuberculosis*, RecR is essential for DNA repair and recombination [32]. For many organisms, the purified protein exists as a dimer in solution and can further assemble into homo-tetramers [51,67,70,73,74]. For EcRecR, the protein exists in a pH-dependent, dimer–tetramer equilibrium [75]. The RecR tetramer is observed in the crystal structures of the Dr- and TeRecO proteins (Figure 3A) [67,70]. The overall structure of the tetramer resembles DNA polymerase processivity factors [67]. Importantly, the central hole in the tetramer, which is 30–35 Å in diameter, is sufficiently large to accommodate duplex DNA [47,67,76].

Tetramer formation is required for RecF and RecO binding [47,75,76]. For the *Deinococcus* proteins, the RecR tetramer binds to two RecO monomers, with one on each side of the ring, obscuring the central hole and preventing access by either DNA or RecF (Figure 3B) [47,61]. In contrast, in the *Thermus* RecFOR–DNA complex, only one RecO monomer is bound to the tetramer face opposite that of RecF (Figure 3C). For both organisms, the OB-fold of each RecO monomer binds to RecR, interacting with the HhH and TOPRIM domains as well as the C-terminal Walker B motif (Figure 2C) [66]. Electrostatic interactions are critical for binding, consistent with regions of positive charge on RecO and the negatively charged surface on the RecR ring faces.

The vast majority of RecF proteins exist as monomers in solution but can form an ATP-induced dimer in the absence of DNA; however, this occurs only at high protein concentrations [46,78]. This is consistent with a low dimerization constant. In contrast, the *Thermoanaerobacter tengcongensis* RecF is monomeric but readily forms a dimer in the presence of either ATP or ATP-γ-S [59].

The structure of the RecF monomer reveals a protein with two domains, namely an N-terminal domain or Lobe I, which includes the Walker A and B motifs, and a C-terminal domain or Lobe II, which is largely α-helical and contains the signature motif (S-motif) first identified in eukaryotic Rad50 and SMC proteins [46,50,59,79,80]. The overall structure of the monomer is very similar to the head domain of SMC proteins and Rad50 but lacks the coiled–coiled domains of the eukaryotic proteins. In the presence of adenine nucleoside triphosphates, RecF forms a homo-dimer containing a cradle, or a DNA binding pocket involving both monomers, that enables the dimer to bind DNA, like a saddle sitting on a horse (Figure 3C) [46,50,59]. In this complex, ATP acts as a fastener linking domain I of monomer one with domain II of monomer two [59]. When bound to ATP and DNA, one face of the RecF dimer interacts with the RecR tetramer, positioning RecO close to the DNA [50].

**Figure 4:**
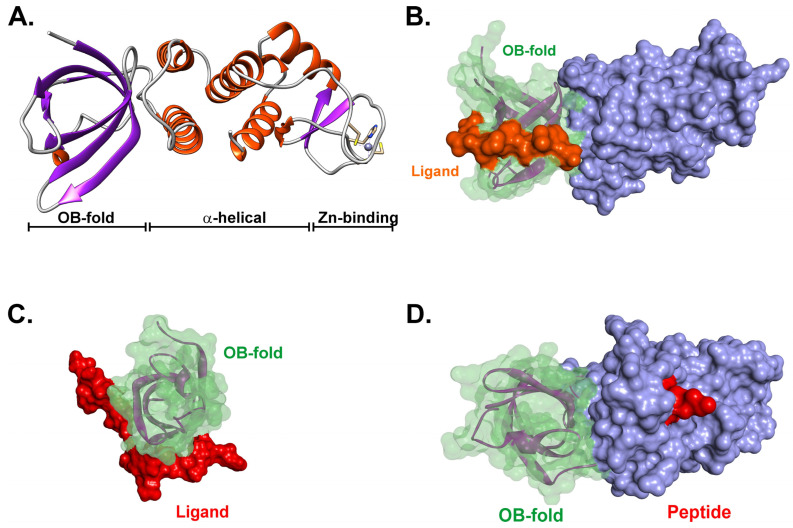
RecO’s structural features are conserved. (**A**) The RecO protein from Campylobacter jejuni (PDB file:7 YMO). The three domains of RecO are indicated. The image was generated using UCSF Chimera [85]. (**B**) An SSB C-terminal peptide binds in the α-helical domain. The structure of the *E.coli* RecO protein bound to a peptide corresponding to the last eight residues is shown. PDB File 3 Q8 D was used to generate the image using Discovery Studio (Biovia). The Connolly surfaces for the OB-fold (green), C-terminal domain (mauve), and peptide (red) are shown [77]. (**C**,**D**) The *E.coli* RecO OB-fold binds to the intrinsically disordered linker of its cognate SSB protein. The OB-fold from 3 Q8 D was aligned with the SH3 domains of PDB files insulin receptor tyrosine kinase substrate 2 XKC (**C**) and abl tyrosine kinase 1 ABO (**D**), respectively [49]. The Connolly surfaces for the OB-fold (green), C-terminal domain (mauve), and PXXP–ligands (red and orange, respectively) are shown [77]. In panel (**C**), the OB-fold/ligand interaction is viewed from the top as in panel (**B**). In (**D**), the RecO–PXXP–ligand complex is viewed from the side.

In contrast to RecF and R, RecO on its own is monomeric, and it is also monomeric when bound to RecR [17,60,64,74]. The crystal structures of the protein from different organisms are generally very similar (Figure 4 and Supplementary Figure S2). There is a well-conserved N-terminal OB-fold, an α-helical domain, and a Cys-4-zinc binding domain [17,34,50,60]. The role of the Zinc binding motif is unknown; however, for *Mycobactrium smegmatis* RecO (MsRecO), ssDNA affinity is enhanced in the presence of zinc [81]. This motif is present in many RecO proteins but is absent in both the *E.coli* and *Thermus thermophilus* proteins.

The RecO OB-fold is a multi-functional domain, enabling separate binding to the RecR tetramer as explained above, with the intrinsically disordered linker of SSB and DNA (Figure 4B,C) [16,61,62,82,83]. It is conceivable that the OB-fold is responsible for regulation, as RecR, SSB, and DNA may bind competitively. The α-helical domain is also involved in binding duplex DNA [61]. In addition, this domain in the *E. coli* protein binds to a short peptide corresponding to the last eight residues of *E. coli* SSB (Figure 4D). However, SSB protein does not require these eight residues to bind RecO, as an SSB deletion mutant (SSBΔC8) still binds RecO [17]. Furthermore, this region in RecO, like many other SSB-binding partners, is poorly conserved, and neither the *M. smegmatis* or *D. radiodurans* RecO proteins bind the *E. coli* SSB peptide, even though the sequence in the cognate proteins is 50% identical [83,84].

## 6. The Mechanism of Action of RecFOR

Following the discovery that the RecF, O, and R proteins are necessary for recombination initiation in the RecF pathway in *E. coli*, they were shown to belong to the same epistatic group [7,8,11,86,87]. Since then, the purified proteins have been characterized independently, together in various combinations, and in the presence of cognate SSB and RecA proteins, consistent with their roles in the early stages of homologous recombination. Studies have focused on protein–protein interactions; DNA binding; DNA cofactor specificity; ssDNA annealing; loading of RecA onto SSB-coated DNA molecules; and the effects on DNA strand exchange mediated by RecA. More recently, RecFOR proteins have been implicated in stalled DNA replication fork rescue and translesion synthesis [88,89,90,91,92,93,94,95,96,97]. Here, RecF and RecR are required to reassemble the replisome following stalling. Please see additional details in [53]. Below, we discuss the activity of each protein, protein complexes, and activities in RecA loading.

### 6.1. RecO and ssDNA Annealing

RecO anneals ssDNA molecules of varying lengths and catalyzes the assimilation of ssDNA into negatively coiled superhelical DNA [17,74,81,84,98,99]. To achieve these outcomes, RecO must bind to ssDNA with reasonable affinity. Consistent K_d_ values ranging from 146 nM to 8.7 µM have been reported (Supplementary Table S1) [51,84,100]. For MsRecO, the addition of zinc increases the affinity of the protein for ssDNA 58-fold [84]. As annealing most likely involves ssDNA coated by SSB protein, the affinity of RecO for both short SSB peptides (the last eight amino acids) and full-length SSB proteins has been measured. Results show that the K_d_ for the peptide ranges from 120–180 nM, while the K_d_ for SSB protein ranges from 7 to 90 nM (Supplementary Table S1) [51,84,100,101]. In annealing reactions with oligonucleotides, the stoichiometry of RecO–ssDNA is low, having an optimum of 1:40 nucleotides (nts). In contrast, in the presence of SSB, this ratio decreases to 1:13 nts, which is almost the same as the SSB–ssDNA stoichiometry of 1:10, resulting in an SSB–RecO ratio of 0.8 [98]. These results indicated that the mode of annealing is different in the absence and presence of SSB. In the absence of SSB, RecO acts via the ssDNA, whereas in its presence, the mediator acts via SSB, consistent with the active species being the RecO–SSB–ssDNA complex.

It is not surprising that the interaction between RecO and single-strand DNA binding proteins is species-specific and essential to the outcome of the reaction [16,84,98,100]. First, eukaryotic replication protein A cannot substitute for SSB. Second, EcRecO binds to EcSSB; *Thermotoga maritima* RecO binds to TmSSB; MsRecO binds to MsSSB; and *Thermus thermophilus* RecO binds to TheSSB. Third, the mechanism of SSB binding to RecO (and other interactome partners) has been a hotly contested issue [82,83,102]. It was initially thought that SSB was bound to RecO and other interactome partners only via its C-terminal eight0 amino acids. This view, however, changed when it was discovered that the intrinsically disordered linker (IDL) of SSB is the primary binding site, docking with the OB-fold in the interactome partner [16]. Deletion of the RecO OB-fold eliminates SSB binding in vivo, and deletion of the SSB IDL eliminates binding to either RecG or RecO [16,62]. Furthermore, even though a peptide corresponding to the last eight residues of EcSSB binds to EcRecO, an SSB mutant lacking the last eight residues still binds to RecO in vitro [17,100]. In *Mycobacteria*, MsRecO does not bind to the tip of MsSSB [81]. Finally, the SSB C-terminal peptide binding pocket in EcRecO is not conserved even though the residues corresponding to the SSB C-terminus are the most highly conserved regions of these proteins [17,83,103,104]. These findings are consistent with the IDL of SSB binding to the OB-fold of RecO forming the primary interaction site (Figure 4B,C) [82,83]. Consistently, the sequences of the IDLs of each SSB are species-specific and, thus, poorly conserved [83,103,104,105]. The role then of the C-terminal 8–10 residues is to regulate the structure of SSB C-terminal tails using long-range electrostatic effects, ensuring they do not bind to SSB OB-folds, as well as to serve as a secondary interaction site stabilizing complex formation [62,83,106].

The interaction of RecO with SSB is critical to the disengagement of the single-strand binding protein from ssDNA and its replacement with RecA, although SSB may not be displaced entirely from the nucleic acid [51,54,81] (Figure 1B–D). The mechanism for how RecO disengages SSB from the ssDNA has been challenging to discern, although some models have been proposed. Inoue et al. proposed that the interaction of RecO with SSB suppresses the dynamics of the mobile C-terminal tail, resulting in a weakening of the affinity of SSB for DNA [100]. Hwang et al. discovered that DrRecO competes with DrSSB for ssDNA binding and takes advantage of the rapid diffusion of the SSB on the ssDNA [107]. When the SSB slides, it makes ssDNA available for DrRecO to bind, ultimately inducing the complete dissociation of SSB from the DNA. They proposed that this occurs via a two-step binding process. In step 1, RecO binds to nascent ssDNA produced by SSB sliding. This forms a heterotrimeric complex of RecO–ssDNA–SSB. In step 2, further interaction between RecO and the DNA occurs, inducing a conformational change in the nucleic acid, resulting in SSB dissociation.

In *E. coli*, the binding of RecO to SSB has been shown to decrease the rate of SSB sliding on ssDNA [108]. Consequently, Bell et al. proposed that instead of taking advantage of rapid sliding, binding of RecO to SSB changes the lifetime of exposed ssDNA (SSB moves slower) and these gaps are then used to initiate annealing and may also function as filament nucleation sites for RecA [109]. For *Thermus* which has a homo-dimeric SSB, the binding of two RecO proteins to the SSB–DNA complex was proposed to lower the affinity of the SSB for the nucleic acid [51]. In addition, the RecOR complex binds to the SSB–ssDNA complex with greater affinity than RecO alone and was proposed to further reduce the affinity of SSB for the nucleic acid, without displacing the homo-dimer from the DNA.

### 6.2. Impact of RecR Binding to RecO

RecR binds to RecO with K_d_ values ranging from 66 to 186 nM (Supplementary Table S2) [64,70,71,100,110]. The impact of this interaction on the affinity of RecO for ssDNA varies from organism to organism. The change in affinity can be attributed to the impact of RecR on RecO as the former does not bind to ssDNA and binds poorly to the DNA duplex [70]. For EcOR, the affinity decreases 6-fold [81,84]. In contrast, for *Thermus*, *Thermoanaerobacter*, and *Deinococcus*, the affinity increases as much as 6-fold [51,70,111]. Surprisingly, the binding of MsRecR to MsRecO increases the affinity of RecO for ssDNA to the same extent that the binding of zinc does; zinc addition to MsOR does not produce a further increase in affinity [81,84].

The binding of RecR to RecO produces the OR complex, which has properties distinct from each protein alone [18,47,54,64,70,109,110,112]. These properties include changes in affinity for ssDNA, inducing further alterations in SSB–ssDNA complexes relative to RecO alone, as well as changes in DNA cofactor specificity and the regulation of RecA nucleoprotein filaments, enhancing nucleation and stabilization. For the *Deinococcus* proteins, the RecR tetramer binds two RecO monomers, whereas proteins from other species bind only one (Figure 3). Molecular modeling was performed to show that within this complex, the OB-fold of RecO can bind to ssDNA [70]. Consistently, the DrRecOR complex shows preferential binding towards the 3′ overhang of an ssDNA–dsDNA hybrid, consistent with a possible role in the rescue of stalled DNA replication forks [47]. In *E. coli*, RecOR suppresses 5′ end-dependent disassembly of RecA from nucleoprotein filaments, resulting in net filament stabilization [64,110]. In addition, RecOR modulates RecA protein function at the 5′ ends of ssDNA, with RecR remaining associated with nascent filaments (RecO was not detected) [112].

### 6.3. The Concerted Actions of RecF, O, and R as RecFOR

RecF binds to ssDNA preferentially, although it is unclear if the binding is cooperative [56,58,113]. The purified protein also inhibits the binding of RecA to ssDNA and coaggregate formation, a key step in homologous recombination in vitro [114]. However, this inhibition is overcome by the presence of RecO and RecR [54]. RecF binds adenosine nucleoside triphosphates, with K_d_ values ranging from 1 to 32 µM, consistent with the protein containing Walker A and B motifs [52,57,59]. The protein hydrolyzes ATP slowly, similar to its structural homolog, Rad50 [46].

Binding to either ss- or dsDNA enhances the affinity of the enzyme for ATP. Similarly, the binding of ATP enhances the affinity of the protein for ss- and dsDNA 36- to 260-fold, respectively (Supplementary Table S3) [59]. Binding to RecR also increases the affinity of RecF for dsDNA and stimulates its weak ATPase activity [58,115]. In addition to binding to RecR, RecF binds to RecO, can form a tripartite complex of RecF–O and -R, and can also bind SSB in the presence of RecO, forming a second tripartite complex of RecF–RecO–SSB [71]. Consistent with the role of RecFOR in loading RecA at post-replication ssDNA gaps, ATP enabled RecF to bind preferentially to gapped DNA, specifically at the ds–ssDNA junction [55]. A later study found that RecF did not show a preference for ss–dsDNA junctions [78]. On DNA cofactors 20–50 bases in length, RecF demonstrates only a modest difference in affinity, and binding was unaffected by RecR [115]. However, the protein exhibits a slight preference for 50 nt circular DNA relative to linear DNA of the same length. Similar results were obtained when binding to model DNAs containing lesions was assessed in the presence and absence of RecR.

**Figure 5:**
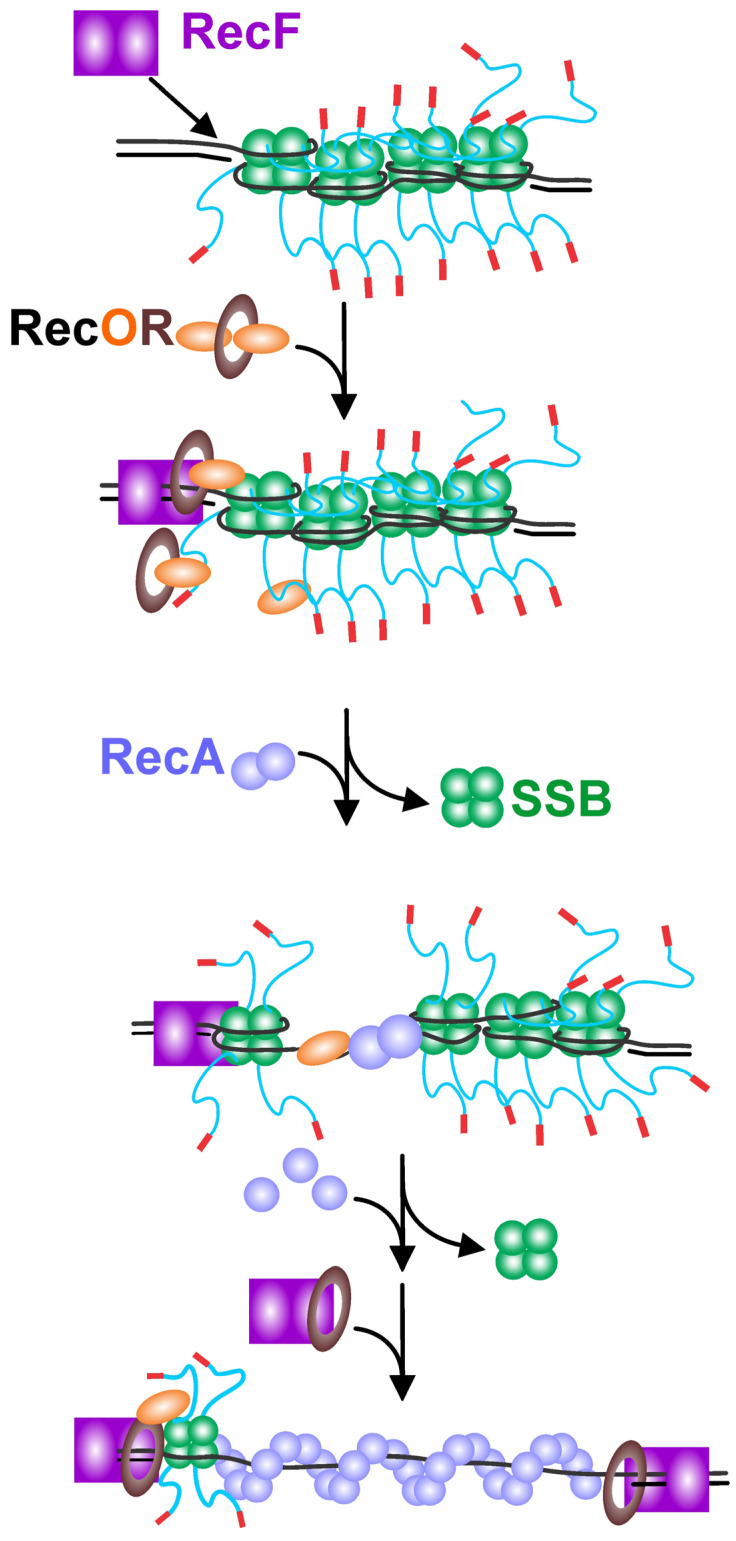
General scheme for RecFOR-mediated loading of RecA onto SSB-coated ssDNA gaps. In the scheme shown, SSB binds to and coats an ssDNA gap. In the first step of RecA loading, the RecF dimer binds to the ss–dsDNA junction. This directs loading of RecOR as either the RecR_4_–RecO_2_ or RecR_4_–RecO complex onto the DNA. This positions RecO in proximity of SSB. The binding of the SSB linker and/or tip to RecO leads to SSB repositioning or dissociation, thereby exposing the ssDNA. RecO may dissociate along with SSB or remain bound to the ssDNA, leaving a RecFR complex at the proximal ss–dsDNA junction. RecA dimers bind to the exposed ssDNA, thereby causing nucleating filament formation. Additional RecA binds extend the filament to coat the ssDNA gap. To limit filament extension into the downstream duplex DNA, a second RecFR complex binds to the distal end of the filament or the ss–dsDNA junction.

In addition to binding ATP and catalyzing very slow hydrolysis, RecF exhibits ATP-dependent dimerization [46,97]. As a dimer, the protein binds to dsDNA like a saddle sitting on top of a horse (Figure 3C and Figure 5). In the presence of ATP and DNA, the RecF dimer binds to the RecR tetramer (possibly in complex with RecO) [72]. The RecFOR complex is most effective when RecF binds at or near an ssDNA–dsDNA junction [12]. This likely results from a network of interactions within the RecFOR complex rather than the direct interaction of RecF with the ss–dsDNA junction [72]. This follows, because neither the RecF dimer nor RecFR complexes bind preferentially to the junction. Thus, it is the complete RecFOR complex that acts as a structure-specific mediator that efficiently directs the loading of RecA onto SSB-coated gapped DNA [13,14,26,28]. Furthermore, it is the collective binding of RecF, O, and R that positions RecO to interact with ssDNA and SSB, which was proposed to lock the complex in position at a ssDNA–dsDNA junction, primed to facilitate RecA nucleoprotein filament nucleation (Figure 5) [50]. However, this is not always the case, as *recF* is not present in *C. jejuni* and H. pylori, where RecA loading is mediated by RecOR [33,34].

Once RecO is positioned by RecR binding, it can then interact with SSB (Figure 5). This interaction, either via ssDNA binding or direct RecO–SSB interactions, results in either SSB dissociation or SSB sliding to vacate the ssDNA. Regardless, the exposed ssDNA is a substrate for RecA filament nucleation by RecA dimers. Nucleation results in rapid filament extension concomitant with SSB displacement. Once the ssDNA gap is covered by an RecA nucleoprotein filament, binding of RecFR to the 3′-end of the filament and/or distal ss–dsDNA junction occurs. This limits the additional extension of RecA into the undamaged duplex DNA region.

## 7. Summary

The RecFOR proteins are widely found in bacteria. They may function as the FR and FOR complexes or as the OR complex in organisms where RecF is absent. While there is a large body of data available providing significant insight into the mechanism of action of these recombination mediators, there is still much to be learned. What is the mechanism of these mediators in the resurrection of stalled DNA replication forks? The in vivo evidence suggests that RecFOR protects stalled replication forks from degradation while enabling the loading of RecA. In addition, these proteins are also proposed to facilitate the reassembly of replisomes. The mechanisms of these fork related activities are completely unknown. What is the mechanism of RecO loading—via SSB interactions with tetramers bound to the DNA, via delivery from a RecO–SSB complex to the SSB bound to the ssDNA or directly to the DNA itself, or via delivery by RecR to the ss–dsDNA junction? Finally, a conundrum that remains to be revealed concerns RecF. If RecFOR is so important for many organisms, why is RecF absent in others? Is there a yet-to-be-identified ortholog to take its place? 

## Figures and Tables

**Table 1:** The structures of RecF, O, and R monomers from different bacteria are very similar.

Protein	Structures Compared ^1^	TM-Align Identity (%)	RMSD (Å)	Host Organisms
RecF	5zwu; 5z67	29	2.29	*Thermus*; *Caldanaerobacter*
	5zwu; 2o5v	40	2.64	*Thermus*; *Deinococcus*
	5z67; 2o5v	32	2.05	*Caldanaerobacter*; *Deinococcus*
RecR	1 vdd; 3 vdu	55	2.17	*Deinococcus*; *Caldanaerobacter*
	3 vdu; 5 z2 v	46	2.18	*Caldanaerobacter*; *Pseudomonas*
	1vdd; 5z2v	48	1.75	*Deinococcus*; *Pseudomonas*
RecO	3 q8 d; 7 ymo	11	3.69	*E. coli*; *Campylobacter*
	3 q8 d; 1 w3 s	13	3.21	*E. coli*; *Deinococcus*
	3 q8 d; 8 ab0	17	2.74	*E. coli*; *Thermus*
	1 w3 s; 8 ab0	29	2.09	*Deinococcus*; *Thermus*
	7 ymo; 8 ab0	13	2.98	*Campylobacter*; *Thermus*

^1.^ The structural alignment was performed using the pairwise structural alignment tool of the RCSB Protein Data Bank [48].

## Supplementary Materials

The following supporting information can be downloaded at: https://www.mdpi.com/article/10.3390/ijms26125441/s1.
