# Supplementary material for: The Role of the RecFOR Complex in Genome Stability
Source: Int J Mol Sci. 2025 Jun 6;26(12):5441. doi: 10.3390/ijms26125441 (PMC12192639; doi:10.3390/ijms26125441)
Supplement: Supplementary file 1 [file ijms-26-05441-s001.zip › ijms-3654731-supplementary.pdf]

Supplementary Material

The Role of the RecFOR Complex in Genome Stability

Piero R. Bianco

Department of Pharmaceutical Sciences, College of Pharmacy, University of Nebraska Medical Center, Omaha, NE 68198-6025, USA; pbianco@unmc.edu

A. RecF

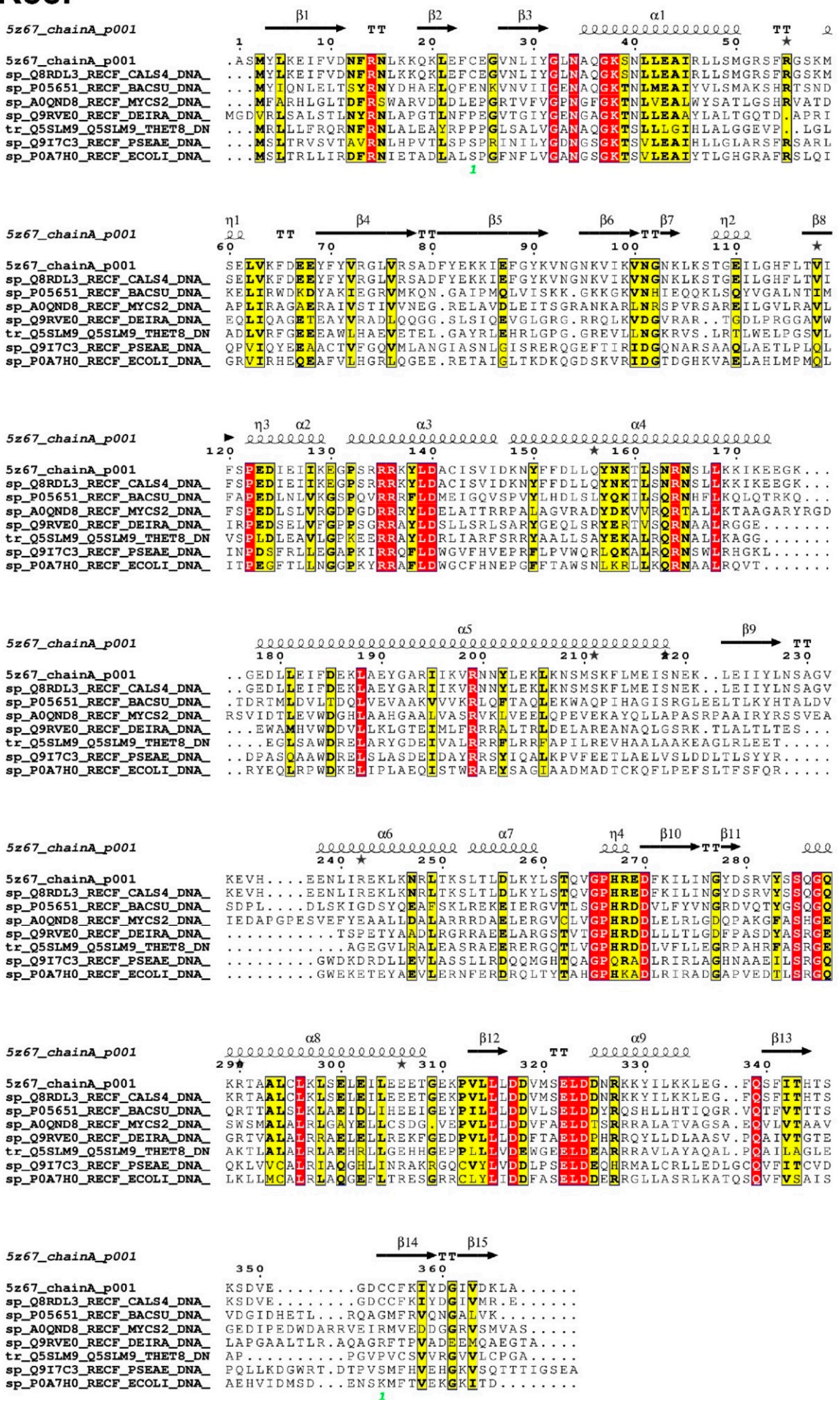
5z67_chainA_p001
5z67_chainA_p001 .ASMYLKEIFVDNFRNLKKQKLEFCEGVNLIYGLNAQGKSNLLEAIRLLSMGRSFRGSKM
sp_Q8RDL3_RECF_CALS4_DNA_ ...MYLKEIFVDNFRNLKKQKLEFCEGVNLIYGLNAQGKSNLLEAIRLLSMGRSFRGSKM
sp_P05651_RECF_BACSU_DNA_ ...MYIQNLELTSYRNYDHAELQFENKVNVIIGENAQGKTNLMEAIYVLSMAKSHRTSND
sp_A0QND8_RECF_MYCS2_DNA_ ...MFARHLGLTDFRSWARVDLDLEPGRTVFVGPNGFGKTNLVEALWYSATLGSHRVATD
sp_Q9RVE0_RECF_DEIRA_DNA_ MGDVRLSALSTLNYRNLAPGTLNFPEGVTGIYGENGAGKTNLLEAAYLALTGQTD.APRI
tr_Q5SLM9_Q5SLM9_THET8_DN ...MRLLLFRQRNFRNLALEAYRPPPGLSALVGANAQGKTSLLGIHLALGGEVP..LGL
sp_Q9I7C3_RECF_PSEAE_DNA_ ...MSLTRVSVTAVRNLHPVTLSPSPRINILYGDNGSGKTSVLEAIHLLGLARSFRSARL
sp_P0A7H0_RECF_ECOLI_DNA_ ...MSLTRLLIRDFRNIETADLALSPGFNFLVGANGSGKTSVLEAIYTLGHGRAFRSLQI
5z67_chainA_p001 SELVKFDEEYFYVRGLVRSADFYEKKIEFGYKVNGNKVIKVNGNKLKSTGEILGHFLTVI
sp_Q8RDL3_RECF_CALS4_DNA_ SELVKFDEEYFYVRGLVRSADFYEKKIEFGYKVNGNKVIKVNGNKLKSTGEILGHFLTVI
sp_P05651_RECF_BACSU_DNA_ KELIRWDKDYAKIEGRVMKQN.GAIPMQLVISKK.GKKGKVNHIEQQKLSQYVGALNTIM
sp_A0QND8_RECF_MYCS2_DNA_ APLIRAGAERAIVSTIVVNEG.RELAVDLEITSGRANKARLNRSPVRSAREILGVLRAVL
sp_Q9RVE0_RECF_DEIRA_DNA_ EQLIQAGETEAYVRADLQQGG.SLSIQEVGLGRG.RRQLKVDGVRAR..TGDLPRGGAVW
tr_Q5SLM9_Q5SLM9_THET8_DN ADLVRFGEEEAWLHAEVETEL.GAYRLEHRLGPG.GREVLLNGKRVS.LRTLWELPGSVL
sp_Q9I7C3_RECF_PSEAE_DNA_ QPVIQYEEAACTVFGQVMLANGIASNLGISRERQGEFTIRIDGQNARSAAQLAETLPLQL
sp_P0A7H0_RECF_ECOLI_DNA_ GRVIRHEQEAFVLHGRLQGEE.RETAIGLTKDKQGDSKVRIDGTDGHKVAELAHLMPMQL
5z67_chainA_p001 FSPEDIEIIKEGPSRRRKYLDACISVIDKNYFFDLLQYNKTLSNRNSLLKKIKEEGK...
sp_Q8RDL3_RECF_CALS4_DNA_ FSPEDIEIIKEGPSRRRKYLDACISVIDKNYFFDLLQYNKTLSNRNSLLKKIKEEGK...
sp_P05651_RECF_BACSU_DNA_ FAPEDLNLVKGSPQVRRRFLDMEIGQVSPVYLHDLSLYQKILSQRNHFLKQLQTRKQ...
sp_A0QND8_RECF_MYCS2_DNA_ FSPEDLSLVRGDPGDRRRYLDELATTRRPALAGVRADYDKVVRQRTALLKTAAGARYRGD
sp_Q9RVE0_RECF_DEIRA_DNA_ IRPEDSELVFGPPSGRRAYLDSLLSRLSARYGEQLSRYERTVSQRNAALRGGE.......
tr_Q5SLM9_Q5SLM9_THET8_DN VSPLDLEAVLGPKEERRAYLDRLIARFSRRYAALLSAYEKALRQRNALLKAGG.......
sp_Q9I7C3_RECF_PSEAE_DNA_ INPDSFRLLEGAPKIRRQFLDWGVFHVEPRFLPVWQRLQKALRQRNSWLRHGKL......
sp_P0A7H0_RECF_ECOLI_DNA_ ITPEGFTLLNGGPKYRRAFLDWGCFHNEPGFFTAWSNLKRLLKQRNAALRQVT.......
5z67_chainA_p001 ..GEDLLEIFDEKLAEYGARIIKVRNNYLEKLKNSMSKFLMEISNEK..LEIIYLNSAGV
sp_Q8RDL3_RECF_CALS4_DNA_ ..GEDLLEIFDEKLAEYGARIIKVRNNYLEKLKNSMSKFLMEISNEK..LEIIYLNSAGV
sp_P05651_RECF_BACSU_DNA_ .TDRTMLDVLTDQLVEVAAKVVVKRLQFTAQLEKWAQPIHAGISRGLEELTLKYHTALDV
sp_A0QND8_RECF_MYCS2_DNA_ RSVIDTLEVWDGHLAAHGAALVASRVKLVEELQPEVEKAYQLLAPASRPAAIRYRSSVEA
sp_Q9RVE0_RECF_DEIRA_DNA_ ...EWAMHVWDDVLLKLGTEIMLFRRRALTRLDELAREANAQLGSRK.TLALTLTES...
tr_Q5SLM9_Q5SLM9_THET8_DN ....EGLSAWDRELARYGDEIVALRRRFLRRFAPILREVHAALAAKEAGLRLEET.....
sp_Q9I7C3_RECF_PSEAE_DNA_ ..DPASQAAWDRELSLASDEIDAYRRSYIQALKPVFEETLAELVSLDDLTLSYYR.....
sp_P0A7H0_RECF_ECOLI_DNA_ ..RYEQLRPWDKELIPLAEQISTWRAEYSAGIAADMADTCKQFLPEFSLTFSFQR.....
5z67_chainA_p001 KEVH....EENLIREKLKNRLTKSLTLDLKYLSTQVGPHREDFKILINGYDSRVYSSQGQ
sp_Q8RDL3_RECF_CALS4_DNA_ KEVH....EENLIREKLKNRLTKSLTLDLKYLSTQVGPHREDFKILINGYDSRVYSSQGQ
sp_P05651_RECF_BACSU_DNA_ SDPL....DLSKIGDSYQEAFSKLREKEIERGVTLSGPHRDDVLFYVNGRDVQTYGSQGQ
sp_A0QND8_RECF_MYCS2_DNA_ IEDAPGPESVEFYEAALLDALARRRDAELERGVCLVGPHRDDLELRLGDQPAKGFASHGE
sp_Q9RVE0_RECF_DEIRA_DNA_ ...........TSPETYAADLRGRRAEELARGSTVTGPHRDDLLLTLGDFPASDYASRGE
tr_Q5SLM9_Q5SLM9_THET8_DN ...........AGEGVLRALEASRAEERERGQTLVGPHRDDLVFLLEGRPAHRFASRGE
sp_Q9I7C3_RECF_PSEAE_DNA_ .........GWDKDRDLLEVLASSLLRDQQMGHTQAGPQRADLRIRLAGHNAAEILSRGQ
sp_P0A7H0_RECF_ECOLI_DNA_ .........GWEKETEYAEVLERNFERDRQLTYTAHGPHKADLRIRADGAPVEDTLSRGQ
5z67_chainA_p001 KRTAALCLKLSELEILEEETGEKPVLLLDDVMSELDDNRKKYILKKLEG..FQSFITHTS
sp_Q8RDL3_RECF_CALS4_DNA_ KRTAALCLKLSELEILEEETGEKPVLLLDDVMSELDDNRKKYILKKLEG..FQSFITHTS
sp_P05651_RECF_BACSU_DNA_ QRTTALSLKLAEIDLIHEEIGEYPILLLDDVLSELDDYRQSHLLHTIQGR.VQTFVTTTS
sp_A0QND8_RECF_MYCS2_DNA_ SWSMALALRLGAYELLCSDG.VEPVLLLDDVFAELDTSRRRALATVAGSA.EQVLVTAAV
sp_Q9RVE0_RECF_DEIRA_DNA_ GRTVALALRRAELELLREKFGEDPVLLLDDFTAELDPHRRQYLLDLAASV.PQAIVTGTE
tr_Q5SLM9_Q5SLM9_THET8_DN AKTLALALRLAEHRLLGEHHGEPPLLLVDEWGEELDEARRRAVLAYAQAL.PQAILAGLE
sp_Q9I7C3_RECF_PSEAE_DNA_ QKLVVCALRIAQGHLINRAKRGQCVYLVDDLPSELDEQHRMALCRLLEDLGCQVFITCVD
sp_P0A7H0_RECF_ECOLI_DNA_ LKLLMCALRLAQGEFLTRESGRRCLYLIDDFASELDDERRGLLASRLKATQSQVFVSAIS
5z67_chainA_p001 KSDVE........GDCCFKIYDGIVDKLA......
sp_Q8RDL3_RECF_CALS4_DNA_ KSDVE........GDCCFKIYDGIVMR.E......
sp_P05651_RECF_BACSU_DNA_ VDGIDHETL...RQAGMFRVQNGALVK........
sp_A0QND8_RECF_MYCS2_DNA_ GEDIPEDWDARRVEIRMVEDDGGRVSMVAS.....
sp_Q9RVE0_RECF_DEIRA_DNA_ LAPGAALTLR.AQAGRFTPVADEEMQAEGTA....
tr_Q5SLM9_Q5SLM9_THET8_DN AP..........PGVPVCSVVRGVVLCPGA.....
sp_Q9I7C3_RECF_PSEAE_DNA_ PQLLKDGWRT.DTPVSMFHVEHGKVSQTTIGSEA
sp_P0A7H0_RECF_ECOLI_DNA_ AEHVIDMSD...ENSKMFTVEKGKITD........

B. RecO

```
                                               β1              β2       β3
tr_Q9RW50_Q9RW50_DEIRA_DN                                    TT         T.T
                            1        10        20        30        40
tr_Q9RW50_Q9RW50_DEIRA_DN  M.......RSRTANRSGIVIRRRVTPAGDIIVTLLTP.QGKLKAI.ARGGVKG..PLSSS
1w3s_chainA_p001           ........RSRTANRSGIVIRRRVTPAGDIIVTLLTP.QGKLKAI.ARG........SSS
sp_A0R0S5_RECO_MYCS2_DNA_  M.........RLYRDRAVVLRQHKLGEADRIVTLLTRDHGLVRAV.AKGVRRTRSKFGAR
sp_Q9XCX7_RECO_PSEAE_DNA_  M........SFAAAQATYVLHSRPYKETSALVDFFTP.LGRLRAV.LRGARGKAG...AL
tr_Q5SKM0_Q5SKM0_THET8_DN  MAPAYTGKVERYRLEEGIVVGRKPLPQGDLLLRLVTP.RGSLEAV.VRKGQRPTGRT.GR
sp_P0A7H3_RECO_ECOLI_DNA_  M..........EGWQRAFVLHSRPWSETSLMLDVFTEESGRVRLV.AKGARSKRSTLKGA
sp_P42095_RECO_BACSU_DNA_  M..........LTKCEGIVLRTNDYGETNKIVTLLTREHGKIGVM.ARGAKKPNSRLSAV
sp_Q8RB48_RECO_CALS4_DNA_  M.........RFLKTEAIVLKSNLISEKDKIATLFTRDYGKLQAV.AKGARRSKSRFVNA
tr_A0AAX0NK37_A0AAX0NK37_  M.............QGFILHTQKVKDEDLIVYILSP.KMLIKAYRFYGLRH......SS
```

```
                                  β4                β5        η1       α1
tr_Q9RW50_Q9RW50_DEIRA_DN     TT              TT
                           50        60        70        80        90       100
tr_Q9RW50_Q9RW50_DEIRA_DN  LNLFHHVGVQVYQG.PHNDLASVKQAVLEGALPTLAEPERYAFAHLMAEFADALFQ.EGE
1w3s_chainA_p001           LNLFHHVGVQVYQG.PHNDLASVKQAVLEGALPTLAEPERYAFAHLMAEFADALFQ.EGE
sp_A0R0S5_RECO_MYCS2_DNA_  LEPFAHIDVQLHPG.RNLDIVTQVQAIDAFAADIVSDYGRYTTACAMLETAERLAGEERA
sp_Q9XCX7_RECO_PSEAE_DNA_  ARPFVPLEAEWRGR.GELKTVARLESAGVPN..LLN.GQALFSGLYLNELLIRLLP.AED
tr_Q5SKM0_Q5SKM0_THET8_DN  LSLFHHVRFQLYAKGEGLPTLTQAELLGRLH..GLEAPRRFLLAAFLAELAYRLAS..PE
sp_P0A7H3_RECO_ECOLI_DNA_  LQPFTPLLLRFGGR.GEVKTLRSAEAVSLAL..PLS.GITLYSGLYINELLSRVLE.YET
sp_P42095_RECO_BACSU_DNA_  SQPFLYGSFLMQKT.SGLGTLQQGEMILSMR.GIREDLFLTAYAAYVAELVDRGTE.EKK
sp_Q8RB48_RECO_CALS4_DNA_  VRPFIVANYVIFEG.QNYYYIDQWELVKNFE.NIEKDLKKFALASYISETISRVLE.EKQ
tr_A0AAX0NK37_A0AAX0NK37_  ILSGYKIDFALEENPSFLPRLKDVLHLGFL..WIMQRDKMLIWQEFIRLLYRHLKD.VEE
```

```
                                  α2                    α3
tr_Q9RW50_Q9RW50_DEIRA_DN                                                  T...TT
                           110       120       130       140       150       160
tr_Q9RW50_Q9RW50_DEIRA_DN  FSEQAFDLFAASLRGVAHQP.DPEWVALVMSYKLLGLAGVIPQTARCA...RCGAPD...
1w3s_chainA_p001           FSEQAFDLFAASLRGVAHQP.DPEWVALVMSYKLLGLAGVIPQTARCA...RCGAPD...
sp_A0R0S5_RECO_MYCS2_DNA_  PMPDLHRLTVAALRAIADGRARELVLDSYLLRAMAIAGWAPALTECA...RCAAPG...
sp_Q9XCX7_RECO_PSEAE_DNA_  PQPEIFAHYAATLPLLAAGR.PIEPLLRAFEWRLLEQLGYGFALDVDI...DGRPIE.PQ
tr_Q5SKM0_Q5SKM0_THET8_DN  AAPRIYPLLVSGLRGIAKHE.DPLLPLVWAGWRVAKAGGIGPNLEGEGLRLKRGRLG...
sp_P0A7H3_RECO_ECOLI_DNA_  RFSELFFDYLHCIQSLAGVTGTPEPALRRFELALLGHLGYGVNFTHCA...GSGEPVDDT
sp_P42095_RECO_BACSU_DNA_  PNPYLFEFILESLKQLNEGT.DPDVITFIVQMKMLGVMGLYPELNHCV...HCKSQD...
sp_Q8RB48_RECO_CALS4_DNA_  KNTKLYFFTKHSLEAVESLNVETSIFLFSYTLKLISLLGYMPVLDSCA...VCGKKE...
tr_A0AAX0NK37_A0AAX0NK37_  LDSFYFDLLDECVKRFEKQ..NPKRVIVDAYLKILEFEGRLHKDFFCF...ACDEKI...
```

```
                                                          α4         α5
tr_Q9RW50_Q9RW50_DEIRA_DN       TTT
                                 170       180       190       200       210
tr_Q9RW50_Q9RW50_DEIRA_DN  .PEHPDPLGGQ.LLC.SKCAALPPY....PPAVLDFLRHAVRRTVRASFEQPVPSADRPA
1w3s_chainA_p001           .PEHPDPLGGQ.LLC.SKCAALPPY....PPAVLDFLRHAVRRTVRASFEQPVPSADRPA
sp_A0R0S5_RECO_MYCS2_DNA_  PHRAFHVAAGG.SVC.VHCRPSGSS..TPPQAVLELMSALHDGDWEYAES..STPPHRSQ
sp_Q9XCX7_RECO_PSEAE_DNA_  ALYQLLPEAGL.EPV.AQLQPGLFQ........GSELLSMADADW.......SAPGALAA
tr_Q5SKM0_Q5SKM0_THET8_DN  .EEGVYLGREGVEALKATLRLPG.....................................AQALPH
sp_P0A7H3_RECO_ECOLI_DNA_  MTYRYREEKGF.IAS.VVIDNKTFT........GRQLKALNAREF.......PDADTLRA
sp_P42095_RECO_BACSU_DNA_  GTFHFSVRDNG.FIC.HRCFEKDPYRIPIKPQTARLLRLFYYFDLSRLGNVSLKEETKAE
sp_Q8RB48_RECO_CALS4_DNA_  NLSYFSSSCGG.AVC.KDCNETCKDAKFLNKKVLKFLLYLLKAKYEKLERISVPGVIKEE
tr_A0AAX0NK37_A0AAX0NK37_  .QNSITLLRAF.LPSHSQCALGFEF.......EEKKLKQFYS....SKNCAIFDDEEIEN
```

```
                                α6            η2
tr_Q9RW50_Q9RW50_DEIRA_DN
                              220       230       240
tr_Q9RW50_Q9RW50_DEIRA_DN  LWRALEKFVTVQVGGVHSWRQLVPSGVPVLS.............................
1w3s_chainA_p001           LWRALEKFVTVQVGGVHSWRQLVP....................................
sp_A0R0S5_RECO_MYCS2_DNA_  ASGLIAAHLQWHLER.....QLRTLPLVERGYRVDRTVAEQRAALVRQDMAHGNHTGQED
sp_Q9XCX7_RECO_PSEAE_DNA_  AKRLMRQALAPHLGGR....PLVSRELFMNRKESPRD.......................
tr_Q5SKM0_Q5SKM0_THET8_DN  LEGAPLNRLFLALKAH....AEEALGPLRSAEAIGV........................
sp_P0A7H3_RECO_ECOLI_DNA_  AKRFTRMALKPYLGGK....PLKSRELFRQFMPKRTV......KTHYE............
sp_P42095_RECO_BACSU_DNA_  LKQVIDLYYEEYSGL.....YLKSKRFLDQMESMKHLMG.....................
sp_Q8RB48_RECO_CALS4_DNA_  ADKIITEYVRTHLEM.....DFKSKDFAMKLSD...........................
tr_A0AAX0NK37_A0AAX0NK37_  LYHLIKEGL...................................................
```

```
tr_Q9RW50_Q9RW50_DEIRA_DN

tr_Q9RW50_Q9RW50_DEIRA_DN  ........
1w3s_chainA_p001           ........
sp_A0R0S5_RECO_MYCS2_DNA_  LPATASGA
sp_Q9XCX7_RECO_PSEAE_DNA_  ........
tr_Q5SKM0_Q5SKM0_THET8_DN  ........
sp_P0A7H3_RECO_ECOLI_DNA_  ........
sp_P42095_RECO_BACSU_DNA_  ..ENKS..
sp_Q8RB48_RECO_CALS4_DNA_  ........
tr_A0AAX0NK37_A0AAX0NK37_  ........
```

C. RecR

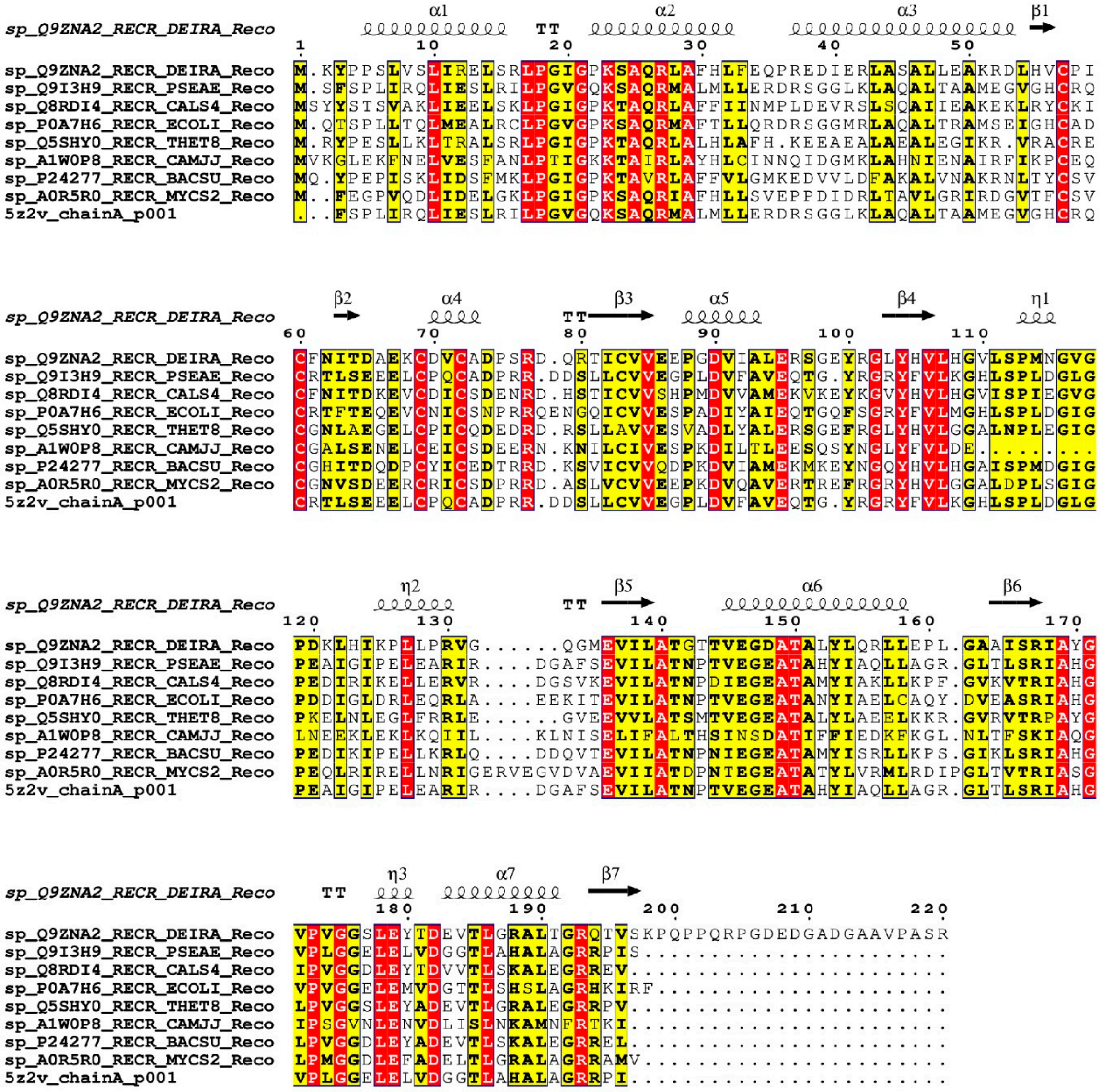

Supplementary Figure S1. The primary amino acid sequences of the RecFOR proteins retain significant similarity. Sequence alignments of the proteins discussed in the text were done using Promals3D and images assembled using ESPript 3 [1-3]. (A). RecF sequences aligned to PDB file 5z67 (*Caldanaerobacter*). The black star above several amino acids indicates critical residues in the alignment. (B), RecO sequences aligned to PDB files 1w3s (*Deinococcus*) and (C), RecR sequences aligned to PDB file 5z2v (*Pseudomonas*). Red boxes, invariant residues. Yellow boxes, highly conserved' TT, beta-turn.

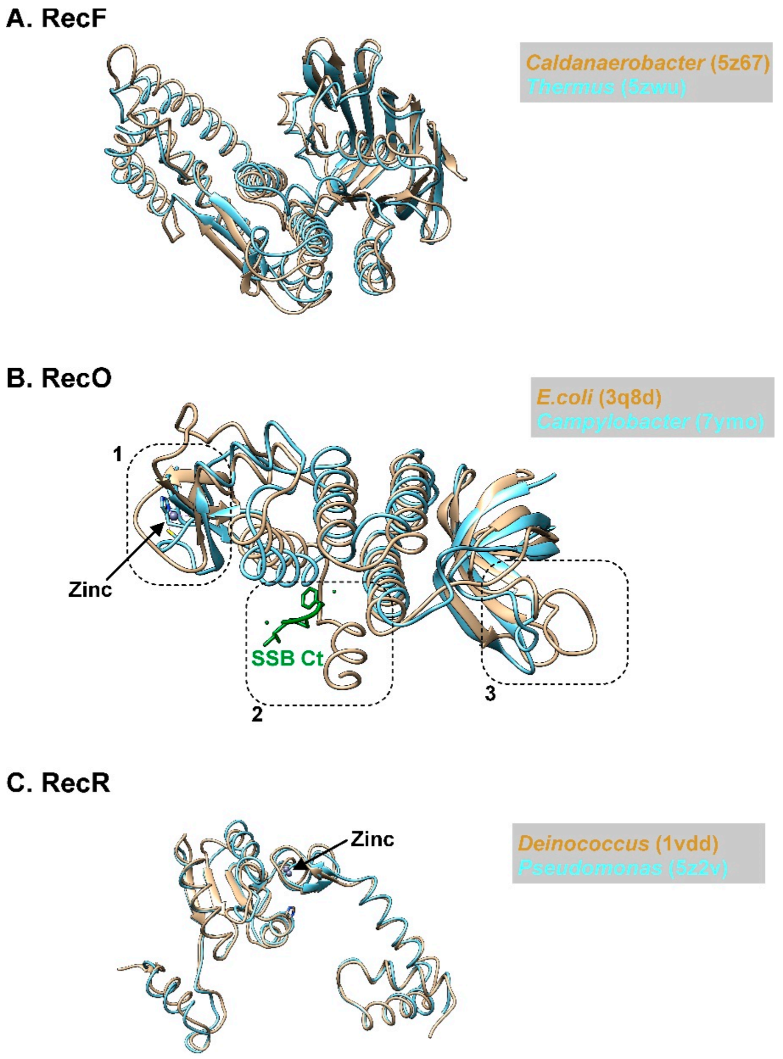

Supplementary Figure S2. The structures of the monomeric forms of RecF, O and R are similar. Structural alignment was done using the monomeric forms of each protein. Alignment was carried out using the structural alignment tool of the PDB, TM-align option [4,5]. The identity of the structures used for comparison are indicated with additional information in Table S1.

Supplementary Table S1. The binding parameters of RecO.

Organism	Interaction	K_d (μM)[1]	Method	Source
E. coli	SSB (Ct)	0.18 ± 0.2	ITC[2]	[6]
	SSB (Ct)	0.12	FA[3]	[7]
	ssDNA	0.45 ± 0.11	FA	[7]
	RecOR – ssDNA	2.76 ± 0.27	FA	[8]
Thermus	SSB	0.0899	SPR[4]	[9]
	ssDNA	binds	EMSA[5]	[9]
	ssDNA	2.4; 0.146	BLI[6]	[10]
	RecR	0.066	SPR	[9]
	RecOR – ssDNA	binds	BLI	[10]
Deinococcus	ssDNA	8.72 ± 0.76	FA	[11]
	RecOR, ssDNA	1.41 ± 0.05	FA	[11]
	RecOR, dsDNA	8.56 ± 0.37	FA	[11]
Mycobacterium	ssDNA	>5<br>2.08 ± 0.11	FA	[7]<br>[8]
	ssDNA (Zn^{2+})	0.036 ± 0.004<br>0.041 ± 0.03	FA	[7]<br>[8]
	RecOR – ssDNA	0.028 ± 0.006	FA	[8]
Thermoanaerobacter	RecR	0.186	SPR	[12]
	RecOR – ssDNA	0.00093	SPR	[12]
	RecOR – dsDNA	0.0264	SPR	[12]

1. Where possible K_d values with published error values are presented. In several instances, only a single value is present in the publication, while in others such as [10], only a "binds" statement is presented.
2. Abbreviations used: 2., ITC, isothermal titration calorimetry; 3., FA, fluorescence anisotropy; 4., SPR, surface plasmon resonance; 5., EMSA, electrophoretic mobility shift assay; and 6., BLI, biolayer interferometry.

Supplementary Table S2. The binding parameters of RecR.

Organism	Interaction	K_d (μM)	Method	Source
Thermoanaerobacter	RecO	0.186	SPR[1]	[12]
	RecR – ssDNA	No interaction	SPR	[12]
	RecR – dsDNA	24.8	SPR	[12]

Abbreviations used: 1., SPR, surface plasmon resonance

Supplementary Table S3. The binding parameters of RecF.

**Organism**	**Interaction[1]**	K_d (µM)	**Method**	**Source**
	**ssDNA**			
E.coli	20mer – 5` FAM	~0.640	FA[2]	[13]
	20mer – 3` FAM	~0.430	FA	[13]
	Oligo $d(T)_{31}$ – 5` FAM	0.093 ± 0.010	FA	[13]
	40mer – 5` FAM	0.134 ± 0.039	FA	[13]
	50mer – iFAM[3]	0.112 ± 0.0238	FA	[13]
	50mer – 5` P – iFAM	0.1157 ± 0.0101	FA	[13]
	50mer circular – iFAM	0.0978 ± 0.022	FA	[13]
	59mer	13	FB	[14]
	**ssDNA + RecR**			
	20mer – 5` FAM	~0.630	FA	[13]
	50mer – iFAM	0.1004 ± 0.0154	FA	[13]
	50mer – 5` P – iFAM	0.1056 ± 0.0195	FA	[13]
	50mer circular – iFAM	0.0684 ± 0.0138	FA	[13]
	**dsDNA**			
	20bp- 5` FAM	0.067 ± 0.01	FA	[13]
	20bp- 3` FAM	0.101 ± 0.0197	FA	[13]
	20bp- 2x 5` FAM	0.139 ± 0.0198	FA	[13]
	20bp- 2x 3` FAM	0.0838 ± 0.0085	FA	[13]
	20bp– 5` +3` FAM	0.0694 ± 0.0613	FA	[13]
	20bp - 2x 5` +3` FAM	0.1473 ± 0.0197	FA	[13]
	20bp– 5` P+5` FAM	0.1338 ± 0.0184	FA	[13]
	31bp	0.110 ± 0.008	FA	[13]
	40bp	0.181 ± 0.059	FA	[13]
	50bp, circular	0.1681 ± 0.0151	FA	[13]
	**dsDNA+RecR**			
	20bp - 5` FAM	0.109 ± 0.014	FA	[13]
	20bp – 5` P+5` FAM	0.1401 ± 0.0115	FA	[13]
	31bp	0.1301 ± 0.0095	FA	[13]
	40bp	0.1738 ± 0.0134	FA	[13]
	50bp, circular	0.2683 ± 0.0332	FA	[13]
Deinococcus	dsDNA	0.25 ± 0.02	FA	[15]
	**Nucleoside**			
Thermoanaerobacter	ATP-γ-S	32	ITC	[16]
	ATP	1.3	ITC	[16]
	ssDNA			
	- ATP	0.576	BLI	[16]
	+ATP	0.016	BLI	[16]
	+ATP-γ-S	0.466	ITC	[16]
	**dsDNA**			
	- ATP	1.63	BLI	[16]

	+ATP	0.0062	BLI	[16]
	+ATP-γ-S	0.381	ITC	[16]

1. Assays with RecF contain ATP, unless indicated otherwise
2. FA, fluorescence anisotropy; FB, filter binding; BLI, biolayer interferometry; ITC, isothermal titration calorimetry
3. iFAM – FAM internally labelled at 25th position of the oligonucleotide

References cited in the Supplementary Material

1. Pei, J.; Grishin, N.V. PROMALS3D: multiple protein sequence alignment enhanced with evolutionary and three-dimensional structural information. *Methods Mol Biol* **2014**, *1079*, 263-271, doi:10.1007/978-1-62703-646-7_17.
2. Pei, J.; Tang, M.; Grishin, N.V. PROMALS3D web server for accurate multiple protein sequence and structure alignments. *Nucleic Acids Res* **2008**, *36*, W30-34, doi:10.1093/nar/gkn322.
3. Pei, J.; Kim, B.H.; Grishin, N.V. PROMALS3D: a tool for multiple protein sequence and structure alignments. *Nucleic Acids Res* **2008**, *36*, 2295-2300, doi:10.1093/nar/gkn072.
4. Bittrich, S.; Segura, J.; Duarte, J.M.; Burley, S.K.; Rose, Y. RCSB protein Data Bank: exploring protein 3D similarities via comprehensive structural alignments. *Bioinformatics* **2024**, *40*, doi:10.1093/bioinformatics/btae370.
5. Zhang, Y.; Skolnick, J. TM-align: a protein structure alignment algorithm based on the TM-score. *Nucleic Acids Res* **2005**, *33*, 2302-2309, doi:10.1093/nar/gki524.
6. Shinn, M.K.; Kozlov, A.G.; Nguyen, B.; Bujalowski, W.M.; Lohman, T.M. Are the intrinsically disordered linkers involved in SSB binding to accessory proteins? *Nucleic Acids Res* **2019**, *47*, 8581-8594, doi:10.1093/nar/gkz606.
7. Gupta, R.; Ryzhikov, M.; Koroleva, O.; Unciuleac, M.; Shuman, S.; Korolev, S.; Glickman, M.S. A dual role for mycobacterial RecO in RecA-dependent homologous recombination and RecA-independent single-strand annealing. *Nucleic Acids Res* **2013**, *41*, 2284-2295, doi:10.1093/nar/gks1298.
8. Ryzhikov, M.; Gupta, R.; Glickman, M.; Korolev, S. RecO protein initiates DNA recombination and strand annealing through two alternative DNA binding mechanisms. *J Biol Chem* **2014**, *289*, 28846-28855, doi:10.1074/jbc.M114.585117.
9. Inoue, J.; Nagae, T.; Mishima, M.; Ito, Y.; Shibata, T.; Mikawa, T. A mechanism for single-stranded DNA-binding protein (SSB) displacement from single-stranded DNA upon SSB-RecO interaction. *J Biol Chem* **2011**, *286*, 6720-6732, doi:10.1074/jbc.M110.164210.
10. Chaudhary, S.K.; Elayappan, M.; Jeyakanthan, J.; Kanagaraj, S. Structural and functional characterization of oligomeric states of proteins in RecFOR pathway. *Int J Biol Macromol* **2020**, *163*, 943-953, doi:10.1016/j.ijbiomac.2020.07.062.
11. Radzimanowski, J.; Dehez F Fau - Round, A.; Round A Fau - Bidon-Chanal, A.; Bidon-Chanal A Fau - McSweeney, S.; McSweeney S Fau - Timmins, J.; Timmins, J. An 'open'

structure of the RecOR complex supports ssDNA binding within the core of the complex.
12. Tang, Q.; Gao, P.; Liu, Y.P.; Gao, A.; An, X.M.; Liu, S.; Yan, X.X.; Liang, D.C. RecOR complex including RecR N-N dimer and RecO monomer displays a high affinity for ssDNA. *Nucleic Acids Res* **2012**, *40*, 11115-11125, doi:10.1093/nar/gks889.
13. Henry, C.; Mbele, N.; Cox, M.M. RecF protein targeting to postreplication (daughter strand) gaps I: DNA binding by RecF and RecFR. *Nucleic Acids Research* **2023**, *51*, 5699-5713, doi:10.1093/nar/gkad311.
14. Griffin, T.J.; Kolodner, R.D. Purification and preliminary characterization of the Escherichia coli K-12 recF protein. *J. Bacteriol.* **1990**, *172*, 6291-6299.
15. Makharashvili, N.; Mi, T.; Koroleva, O.; Korolev, S. RecR-mediated modulation of RecF dimer specificity for single- and double-stranded DNA. *J Biol Chem* **2009**, *284*, 1425-1434, doi:10.1074/jbc.M806378200.
16. Tang, Q.; Liu, Y.P.; Shan, H.H.; Tian, L.F.; Zhang, J.Z.; Yan, X.X. ATP-dependent conformational change in ABC-ATPase RecF serves as a switch in DNA repair. *Sci Rep* **2018**, *8*, 2127, doi:10.1038/s41598-018-20557-0.
